# Immune Priming and the Risk of COVID‐19, Influenza, and Other Acute Respiratory Infections: Insights From an N3C Cohort

**DOI:** 10.1111/irv.70253

**Published:** 2026-03-23

**Authors:** Tomás M. León, Lyndsey M. Muehling, Rachel Baccile, Olga Morozova, National Clinical Cohort Collaborative (N3C) COVID‐19 Consortium, Christopher G. Chute, Adam B. Wilcox, Adam M. Lee, Alexis Graves, Alfred Anzalone, Amin Manna, Amit Saha, Amy Olex, Andrea Zhou, Andrew E. Williams, Andrew M. Southerland, Andrew T. Girvin, Anita Walden, Anjali Sharathkumar, Benjamin Amor, Benjamin Bates, Brian Hendricks, Brijesh Patel, G. Caleb Alexander, Carolyn T. Bramante, Cavin Ward‐Caviness, Charisse Madlock‐Brown, Christine Suver, Christopher G. Chute, Christopher Dillon, Chunlei Wu, Clare Schmitt, Cliff Takemoto, Dan Housman, Davera Gabriel, David A. Eichmann, Diego Mazzotti, Donald E. Brown, Eilis Boudreau, Elaine L. Hill, Emily Carlson Marti, Emily R. Pfaff, Evan French, Farrukh M. Koraishy, Federico Mariona, Fred Prior, George Sokos, Greg Martin, Harold P. Lehmann, Heidi Spratt, Hemalkumar B. Mehta, J. W. Awori Hayanga, Jami Pincavitch, Jaylyn Clark, Jeremy Richard Harper, Jessica Yasmine Islam, Jin Ge, Joel Gagnier, Johanna J. Loomba, John B. Buse, Jomol Mathew, Joni L. Rutter, Julie A. McMurry, Justin Guinney, Justin Starren, Karen Crowley, Katie Rebecca Bradwell, Kellie M. Walters, Ken Wilkins, Kenneth R. Gersing, Kenrick Cato, Kimberly Murray, Kristin Kostka, Lavance Northington, Lee Pyles, Lesley Cottrell, Lili M. Portilla, Mariam Deacy, Mark M. Bissell, Marshall Clark, Mary Emmett, Matvey B. Palchuk, Melissa A. Haendel, Meredith Adams, Meredith Temple‐O’Connor, Michael G. Kurilla, Michele Morris, Nasia Safdar, Nicole Garbarini, Noha Sharafeldin, Ofer Sadan, Patricia A. Francis, Penny Wung Burgoon, Philip R. O. Payne, Randeep Jawa, Rebecca Erwin‐Cohen, Rena C. Patel, Richard A. Moffitt, Richard L. Zhu, Rishikesan Kamaleswaran, Robert Hurley, Robert T. Miller, Saiju Pyarajan, Sam G. Michael, Samuel Bozzette, Sandeep K. Mallipattu, Satyanarayana Vedula, Scott Chapman, Shawn T. O’Neil, Soko Setoguchi, Stephanie S. Hong, Steven G. Johnson, Tellen D. Bennett, Tiffany J. Callahan, Umit Topaloglu, Valery Gordon, Vignesh Subbian, Warren A. Kibbe, Wenndy Hernandez, Will Beasley, Will Cooper, William Hillegass, Xiaohan Tanner Zhang

**Affiliations:** ^1^ California Department of Public Health Richmond California USA; ^2^ Division of Asthma, Allergy & Immunology University of Virginia School of Medicine Charlottesville Virginia USA; ^3^ Center for Health and Social Sciences University of Chicago Chicago Illinois USA; ^4^ Department of Public Health Sciences University of Chicago Chicago Illinois USA

**Keywords:** COVID‐19 vaccines, crossreactive immunity, influenza vaccines, respiratory viruses, SARS‐CoV‐2, vaccination, viral interference

## Abstract

**Background:**

The emergence of SARS‐CoV‐2 and introduction of COVID‐19 vaccines into immunologically naïve populations may alter the dynamics of other acute viral respiratory infections (viral ARIs) and vice versa. Competing forces, including viral interference, cross‐reactive immunity, shared susceptibility, and immune dysregulation, may affect the risk. The potential net impact of various immune‐priming events and their timing on the risk of viral ARIs is largely unknown.

**Methods:**

Using data from the National Clinical Cohort Collaborative (N3C) COVID‐19 Enclave, this retrospective population‐based cohort study investigated the relationship between immune‐priming events (COVID‐19 and influenza vaccinations, and SARS‐CoV‐2, influenza, other, and unspecified viral ARIs) between January 2018 and September 2021 and the risk of viral ARIs during October 2021–April 2022. The sample included *N* = 608,725 individuals from seven data partners with well‐ascertained COVID‐19 and influenza vaccination data.

**Results:**

Early COVID‐19 vaccination (December 2020–March 2021) and SARS‐CoV‐2 infection during the overlapping period (October 2020–March 2021) were associated with a lower risk of all outcomes, including non‐SARS‐CoV‐2 infections. Off‐season influenza vaccination (January–June 2021) was associated with a lower risk of SARS‐CoV‐2 and any viral ARI. Other priming events showed mixed associations, with a lack of evidence of stronger protection from more recent immune‐priming events.

**Conclusions:**

This exploratory analysis suggests potential crossprotection between viral ARIs that may inform vaccination strategies. While ascertainment and healthcare‐seeking biases in electronic health records may inflate positive associations between infection outcomes and immune priming, negative (i.e., protective) associations are of potential public health significance and warrant further investigation.

## 1 Introduction

The COVID‐19 pandemic significantly altered human behavior and social contact patterns for over 2 years, disrupting the normal trends of seasonal respiratory virus circulation and consequently population‐level immunity and burden [1]. After several years of reduced influenza and respiratory syncytial virus (RSV) activity in the United States, both pathogens returned to pre‐COVID‐19 burden levels in 2022–23, with a notably early influenza season and a surge in pediatric hospitalizations for RSV [2, 3]. As SARS‐CoV‐2 becomes an endemic infection with transmission driven by factors such as viral evolution and human behavior [4], it will also be important to understand how recent SARS‐CoV‐2 infection and vaccination impact individual‐level susceptibility to diverse respiratory viruses, and vice versa.

Antiviral immunity is complex and varies according to the timing and context of inciting exposures (infection and/or vaccination). Within a short time frame, an acute viral respiratory infection (viral ARI) may protect the host from subsequent infection with another pathogen (viral interference) [5, 6, 7, 8, 9], or else render the host susceptible to coinfection or superinfection [10, 11]. Long‐term impacts may include induction of crossreactive immunity [12, 13, 14, 15, 16], nonspecific “boosting” of immunity [17, 18], and/or host priming for enhanced response [19]. In contrast, immune imprinting and “original antigenic sin” might negatively impact an individual's ability to respond to future exposures [20, 21, 22]. Sustained immune dysregulation after SARS‐CoV‐2 or influenza infection has also been described [23, 24, 25, 26, 27], but impacts on long‐term viral ARI susceptibility are unclear. These dynamics are further complicated by potential shared susceptibility across viruses, as has been suggested for SARS‐CoV‐2 and influenza [28]. This complexity is highlighted in the influenza vaccine literature, where studies report apparent increased risk [29, 30, 31], no change in risk [32, 33, 34], or decreased risk [35] for noninfluenza ARIs following influenza vaccination, potentially influenced by age, season, and alterations in temporary nonspecific immunity. Here, we leverage the unique situation of SARS‐CoV‐2's emergence into a naive population, its widespread transmission and consequent rapid vaccination response, and extensive SARS‐CoV‐2 infection and vaccination data to interrogate individual‐level risk for viral ARIs following exposure to various immune‐priming events in a population‐based cohort, providing new insight into the potential complex factors that contribute to infection risk.

## 2 Methods

### 2.1 Study Design and Setting

The analyses described in this retrospective population‐based cohort study were conducted with data or tools accessed through the NCATS National Clinical Cohort Collaborative (N3C) COVID‐19 Data Enclave https://n3c.ncats.nih.gov/clinical‐cohort and N3C Attribution & Publication Policy v 1.2‐2020‐08‐25b supported by NCATS Contract No. 75N95023D00001 and Axle Informatics Subcontract: NCATS‐P00438‐B. This research was possible because of the patients whose information is included within the data and the organizations [36] and scientists who have contributed to the ongoing development of this community resource [37].

The N3C aggregates and harmonizes EHR data for patients with laboratory‐confirmed or suspected COVID‐19 during any encounter (including both inpatient and outpatient visits) after January 1, 2020 from data partners (i.e., sites) across the U.S. COVID‐19‐positive cases are matched to COVID‐19‐negative controls at a ratio of 1:2. Sites are asked to upload 2 years of health histories before the earliest COVID‐19 test date for each patient and all records after that date. The design, sampling, and harmonization methods used in the N3C Enclave have been described previously [37, 38]. This study used the N3C Limited Data Set (LDS), which retained dates of clinical services without shifting [39].

### 2.2 Study Population

**FIGURE 1:**
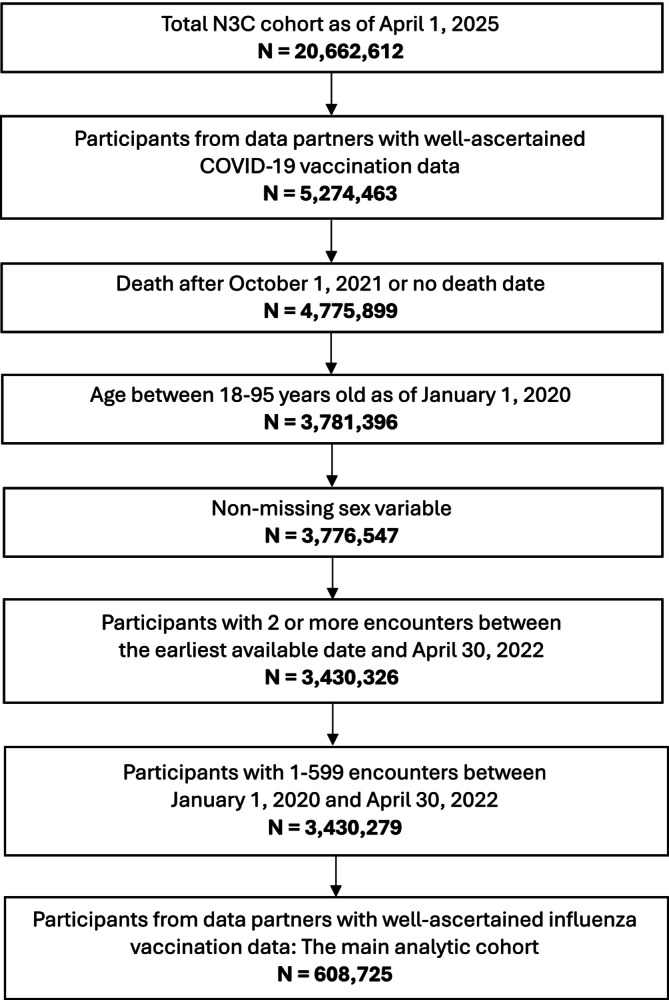
Analytic cohort definition flowchart: number of participants meeting the study inclusion criteria.

The study included all adult (age 18–95) patients in the N3C Enclave (as of April 1, 2025; Release‐v189‐2025‐03‐13) who were alive as of October 1, 2021, and who had two or more health encounters between the earliest available date and April 30, 2022. We further excluded patients with missing sex information and those with zero or above 599 encounters during January 1, 2020–April 30, 2022. Due to limited overall reliability of vaccination data in EHR datasets, we restricted our sample to data partners with well‐ascertained COVID‐19 vaccination data as previously defined. Among the 12 data partners identified by the authors of a previous study as having reliable COVID‐19 vaccination data [40], we further restricted our analysis to data partners with well‐ascertained influenza vaccination data, defined as at least 25% average influenza vaccination coverage during the 2018–19 and 2019–20 influenza seasons (Table S1), resulting in the final sample size of *N* = 608,725 across seven sites. Figure 1 shows the breakdown of the analytic sample inclusion criteria.

### 2.3 Exposures and Outcomes

**FIGURE 2:**
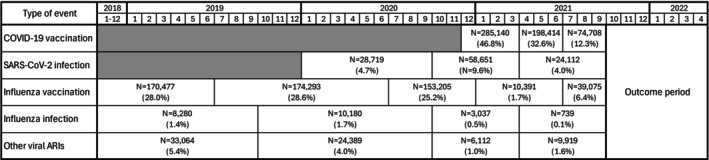
Temporal binning of immune‐priming events in relation to the outcome period. Data in cells show sample counts and proportions corresponding to each immune‐priming event (*N* = 608,725). Numbers in the figure header represent calendar years and months.

Immune‐priming events (exposures) were defined as COVID‐19 vaccination, SARS‐CoV‐2 infection, seasonal influenza vaccination, seasonal influenza infection, and other viral ARIs (including RSV, other specified viral respiratory infections, and unspecified viral respiratory infections) occurring between the earliest available date (generally 2018) and September 30, 2021. These exposures were identified in the N3C Enclave using N3C standard definitions (COVID‐19 vaccination, SARS‐CoV‐2 infection) and concept sets created by the study team (seasonal influenza vaccination, seasonal influenza infection, RSV, other specified and unspecified viral respiratory infections). In the definitions of “other ARIs,” we chose to be inclusive of various infections that were not specifically SARS‐CoV‐2 or influenza. Due to the possibility of superinfection or secondary infection, some of the infections included in these concept sets may have a nonviral etiology. Table S2 provides N3C URLs for each of the concept sets, which describe the positive laboratory tests, diagnosis codes, and other elements that make up the concept definition. These concept sets are also available outside of the N3C Enclave via the GitHub repository: https://github.com/olyamorozova/N3C‐ARI‐concepts. Each exposure type was binned into time periods based on the hypothesis that priming events with temporal proximity to the outcome may exhibit more granular and substantial effects. Figure 2 illustrates the approach. COVID‐19 vaccination was categorized into three quarterly bins with December 2020 merged into the first bin (limited numbers of vaccines were administered in December 2020). SARS‐CoV‐2 infection was categorized into three bins with the first bin covering the initial waves (9 months), and subsequent 6‐month bins. Influenza vaccination was categorized using annual binning (July–June to cover vaccination provided for each standard influenza season) for 2018–19 and 2019–20, 6‐month binning during the 2020–21 influenza season, and 3‐month binning for July–September 2021. Influenza, other, and unspecified respiratory infections were categorized according to each respiratory virus season (October–September) with 6‐month binning during the most recent year.

We analyzed the following infection outcomes occurring between October 1, 2021–April 30, 2022: (1) Any viral ARI (SARS‐CoV‐2, influenza, other/unspecified); (2) Any viral ARI excluding SARS‐CoV‐2; (3) SARS‐CoV‐2 infection; and (4) seasonal influenza infection.

All exposure and outcome events were treated as binary variables and analyzed independently. For variables that aggregated multiple event types (e.g., “any viral ARI”), the analysis did not differentiate between individuals who experienced a single event and those who experienced multiple events.

### 2.4 Statistical Analysis

In descriptive analyses, continuous variables are reported as mean and standard deviation (SD) and categorical variables as number and percentage of patients. If the count of patients was fewer than 20, the number and percentage were not reported per N3C privacy policy.

Correlates of infection outcomes were analyzed using logistic regression with cluster‐robust standard errors to account for potential within‐site correlation [41]. In addition to all immune‐priming events, covariates in regression models included age (under 65, ≥ 65 years of age), sex, race/ethnicity, Charlson Comorbidity Index [42] (CCI: 0, 1–2, ≥ 3) calculated using the N3C Enclave template (Table S2); the log of the number of healthcare encounters between January 1, 2020 and April 30, 2022, and fixed effects for site. All multivariable regression models included the complete set of covariates; no automated subset selection methods were employed.

The study was conducted in accordance with the Strengthening the Reporting of Observational Research Studies in Epidemiology (STROBE) guidelines [43] using Palantir Foundry (Palantir Technologies Inc.) Python version 3.9 and R version 4.0.2 hosted within the N3C Enclave.

#### 2.4.1 Sensitivity and Subcohort Analyses

In addition to the main analysis conducted using the sample from seven data partners, in sensitivity analysis we analyzed a subsample of *N* = 213,759 patients from four data partners with “highly ascertained” influenza vaccination data (≥50% of the data partner cohort receiving a documented influenza vaccination on average during 2018–19 and 2019–20 influenza seasons; Table S1).

### 2.5 Ethics Statement

This study was approved by the institutional review boards at The University of Chicago (#IRB24‐0682), and the NCATS N3C Data Access Committee (DUR‐8544401). The N3C data transfer to NCATS is performed under a Johns Hopkins University Reliance Protocol # IRB00249128 or individual site agreements with NIH. The N3C Data Enclave is managed under the authority of the NIH; information can be found at https://ncats.nih.gov/n3c/resources. The institutional review board at the University of Virginia and the Committee for the Protection of Human Subjects at the State of California Health and Human Services Agency determined this study as exempt from review.

## 3 Results

The main analytic cohort included 608,725 adults (mean [SD] age, 47.0 [18.4] years old) from seven data partners with well‐ascertained vaccination data. The sample was predominantly White (62.8%), a slight majority was female (57.5%), and approximately half had no reported comorbidities (51.1%) (Table 1).

**TABLE 1:** Characteristics of the main analytic cohort (*N* = 608,725).

Characteristic	No. (%) / Mean (SD)
Age (years old): No. (%)	
Less than 65	481,987 (79.2)
65 and older	126,738 (20.8)
Age (years old): Mean (SD)	47.0 (18.4)
Sex: No. (%)	
Male	258,441 (42.5)
Female	350,284 (57.5)
Race/Ethnicity: No. (%)	
Non‐Hispanic White	382,425 (62.8)
Non‐Hispanic Black	32,361 (5.3)
Non‐Hispanic Asian	38,284 (6.3)
Non‐Hispanic other	20,937 (3.4)
Hispanic or Latino, any race	89,005 (14.6)
Unknown	45,713 (7.5)
Charlson Comorbidity Index: No. (%)	
0	311,051 (51.1)
1–2	156,676 (25.7)
3 or greater	140,998 (23.2)
Number of encounters between January 1, 2020 and April 30, 2022: Mean (SD)	23.4 (29.6)
Data partner: No. (%)^a^	
DP 1	92,004 (15.1)
DP 2	62,167 (10.2)
DP 3	31,324 (5.1)
DP 4	28,264 (4.6)
DP 5	166,840 (27.4)
DP 6	124,363 (20.4)
DP 7	103,763 (17.0)

^a^ The N3C data partner identifiers were masked with an arbitrary identifier per N3C data privacy policy.

The prevalence of immune‐priming events of interest (period ending September 30, 2021) is summarized in Figure 2 and Table S3. About 47% of the sample had a record of early COVID‐19 vaccination during December 2020–March 2021, and rates declined in later months. Influenza vaccination coverage was between 25% and 29% for previous seasons (2018–19, 2019–20, 2020–21), partially conditioned by the inclusion criteria. Infection incidence was highest for SARS‐CoV‐2 (with about 10% during October 2020–March 2021). Table 2 shows the cumulative incidence of viral ARI outcomes of interest during October 1, 2021–April 30, 2022. Approximately 14% of the sample had a record of at least one ARI during this period, with the majority being SARS‐CoV‐2 (about 11% of the sample).

**TABLE 2:** Respiratory viral infection outcomes during October 1, 2021–April 30, 2022 (*N* = 608,725).

Outcome	No. (%)
Any respiratory viral infection	82,986 (13.6)
Any respiratory viral infection excluding SARS‐CoV‐2	23,146 (3.8)
SARS‐CoV‐2 infection	64,715 (10.6)
Influenza infection	4704 (0.8)

**FIGURE 3:**
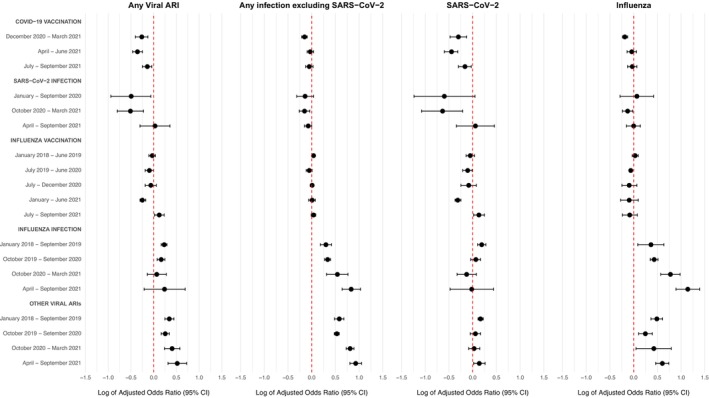
Multivariable logistic regression results for the correlates of acute respiratory infection outcomes during October 1, 2021–April 30, 2022 (*N* = 608,725). All regression models are adjusted for age, sex, race/ethnicity, Charlson Comorbidity Index, data partner, and log of the number of healthcare encounters between January 1, 2020–April 30, 2022. Regression coefficients and respective 95% confidence intervals are plotted on the natural log scale. Numeric regression results on the original scale are provided in Table S4.

Multivariable logistic regression for the correlates of infection outcomes revealed several interesting associations (Figure 3, Table S4). COVID‐19 vaccination during December 2020–March 2021 was correlated with a lower risk of all infection outcomes, whereas COVID‐19 vaccination during later periods was significantly correlated with a lower risk of SARS‐CoV‐2, but not influenza or other non‐SARS‐CoV‐2 infections.

Early SARS‐CoV‐2 infection during January–September 2020 was correlated with a lower risk of any viral ARI but not significantly associated with any of the specific infection outcomes. SARS‐CoV‐2 infection during October 2020–March 2021 was correlated with lower risk of all outcomes. By contrast, more proximal SARS‐CoV‐2 infection (during April–September 2021) was not significantly associated with any outcome.

Off‐season influenza vaccination during January–June 2021 was correlated with a lower risk of SARS‐CoV‐2 and any ARI, with mostly nonsignificant results for influenza vaccinations during other periods and for other outcomes. Influenza infection and other viral ARIs were either nonsignificant or associated with an increased risk of all outcomes of interest.

In sensitivity analysis, results of multivariable regression analyses in a subsample of four sites with “highly ascertained” influenza vaccination data are shown in Table S5. Most of the results remain qualitatively similar, with some associations becoming nonsignificant. The association between off‐season influenza vaccination during January–June 2021 and lower risk of SARS‐CoV‐2 and any viral ARI remains significant in this analysis. In addition, both off‐season influenza vaccination (January–June 2021) and early season influenza vaccination (July–September 2021) appear to be associated with significantly lower risk of influenza infection during October 2021–April 2022.

## 4 Discussion

This analysis used the extensive N3C dataset to answer novel questions around the potential effects of COVID‐19 and influenza vaccination and prior infections on the risk of future respiratory virus infections. With over 600,000 patients from seven sites in the main analytic cohort, the analysis leveraged the unique strengths of N3C for exploratory investigation. The results show both well‐documented findings and reveal interesting novel associations that suggest possible cross‐virus protection and require more targeted analyses.

Similar to previous findings [44], COVID‐19 vaccination was consistently associated with reduced risk of subsequent SARS‐CoV‐2 infection documented through N3C (October 2021–April 2022), although not always significant in subcohort analysis. The protective association was strongest for COVID‐19 vaccines administered during April–June 2021 and weakest for those administered during July–September 2021. The somewhat lower effectiveness of the earlier COVID‐19 vaccination (December 2020–March 2021) could be related to waning immunity [45]. The apparent reduction of protection from the later vaccination (July–September 2021) could be related to the increasing incidence of prior SARS‐CoV‐2 infection over time with moderate additional protection from the vaccine [46] and potentially higher social contact among recently vaccinated people [47].

Notably, early COVID‐19 vaccination (December 2020–March 2021) was modestly associated with reduced risk of influenza and other non‐SARS‐CoV‐2 respiratory infections. This association diminished for vaccinations administered in later months. While it is not possible to distinguish a biological effect of COVID‐19 vaccination from it serving as a proxy for other unmeasured health‐protective behaviors [48], a similar protective association was observed for individuals with documented SARS‐CoV‐2 infection during the overlapping period (October 2020–March 2021). Given that infection may correlate with unmeasured risk‐taking behaviors or higher exposure intensity, the parallel protective effects seen for both vaccination and infection support the possibility of a biological effect of immune priming during this temporal window.

Influenza vaccination in the 2019–20 and 2020–21 seasons was modestly protective against influenza infection in the outcome period and showed mixed associations with respect to SARS‐CoV‐2 and other respiratory infections. An interesting and potentially important association was seen in those who received influenza vaccine during January–June 2021. This relatively small group (1.7% of total cohort; 2.7% of subcohort with the majority vaccinated during January–March) had a significantly lower risk of any viral ARI, SARS‐CoV‐2, and influenza infection during the outcome period–an effect that remained significant in the sub‐analysis of four sites with highly ascertained influenza vaccination data. The reasons for receiving an off‐season influenza vaccination may include job requirements, pregnancy, travel, immunocompromised status, and other atypical situations. In some cases, off‐season influenza vaccine could be given off‐label as a second dose to patients deemed at higher risk of influenza. While this practice is not explicitly recommended by the Advisory Committee on Immunization Practices, 12% (1270/10,391) of the study participants who received influenza vaccine during January–June 2021 had a record of influenza vaccination during July–December 2020. In the subset of four data partners with highly ascertained vaccination data, this proportion was 17% (1008/5863). The potential protective effect of off‐season influenza vaccination against SARS‐CoV‐2 and other viruses is of interest and requires further investigation.

Prior SARS‐CoV‐2 infection was broadly found to be protective against any respiratory infection, with the strongest effects against SARS‐CoV‐2 among those with documented infection in 2020–early 2021, but not in the most recent 6 months before the outcome period. This finding does not directly agree with extensive literature on SARS‐CoV‐2 immunity and its waning, which would predict a stronger protective effect of more recent exposures [49, 50, 51]. We posit that recent SARS‐CoV‐2 infection could lead to a higher sense of security and thus more social contact during late 2021–early 2022 [47, 52], when non‐pharmaceutical intervention use was continuing to decline [53, 54]. The outcome period encompasses the emergence and dominance of Omicron (BA.1 and BA.2 waves), which changed the nature of protection from both prior vaccinations and infections due to increased immune escape [45, 55, 56]. In addition to potentially higher social contact among those with a more recent infection, the protective effects observed for an earlier SARS‐CoV‐2 infection may be related to changing characteristics of people who experienced their first infection during different epidemic waves. Early infections were more likely among essential workers [57] who, on average, are healthier than the general population, were more likely to be vaccinated earlier, and perhaps more likely to experience asymptomatic or mild SARS‐CoV‐2 infection that was not reported to N3C during the outcome period. Early infections among vulnerable individuals, including those in congregate settings, were less likely to appear in our sample due to the sample inclusion criteria (survival through October 2021) and limited representation of congregate settings like prisons and nursing homes in N3C. In addition, most infections in 2020–early 2021 occurred before COVID‐19 vaccines were available. Such individuals could benefit from hybrid immunity associated with vaccination following infection. By contrast, those who were infected during April–September 2021 may carry “post‐breakthrough infection” immunity (infection following vaccination), raising questions about the possible effect of order of both types of exposure on immunity. Finally, SARS‐CoV‐2 transmission during April–September 2021 was dominated by the Delta variant, which was associated with more severe disease [58], and could lead to weakening of the immune system as opposed to serving as protective immune priming.

Prior influenza infection was associated with a higher likelihood of documented infection for all respiratory infections. Because individuals generally have many influenza infections over the course of their lifetimes, this association may indicate a predisposition to more severe disease and therefore a higher likelihood of contact with the healthcare system for symptomatic influenza. This same finding was generally demonstrated for the history of other non‐SARS‐CoV‐2, noninfluenza respiratory infections, suggesting these records are a proxy for increased susceptibility, risk of severe disease and/or healthcare seeking, independent of risk captured by covariates like CCI and the frequency of encounters.

### 4.1 Strengths and Limitations

An important limitation of our study is inherent to all analyses of EHR data, where reporting is incomplete to an unknown degree, leading to misclassification of exposures and outcomes [59, 60]. In the presence of ascertainment bias under our study design, positive associations may not necessarily reflect the true elevated risk but rather healthcare‐seeking or other similar behavior [61, 62], which makes reporting of an event predictive of future records of similar events. Our regression adjustment strategy, in particular adjustment for CCI and healthcare encounter frequency, aimed to minimize ascertainment bias but is unlikely to eliminate it. In this setting, evidence of negative associations (i.e., protective effects) deserves more trust and is likely an underestimate of the true effect size. We therefore focus our attention on the potential promising crossprotective associations observed for immune‐priming events, in particular SARS‐CoV‐2 and influenza vaccinations, which warrant further investigation. One possible explanation for the observed crossprotective effects is a positive correlation in individuals' propensity to receive different types of vaccines [63]. Although our analyses were restricted to data partners with well‐ascertained vaccination records, some misclassification of vaccination status remains possible. While such misclassification could bias estimated crossprotective associations away from the null, it is unlikely to fully account for our findings, given the heterogeneity of crossprotective effects observed across exposure time bins despite the persistence of correlated vaccination behavior over time.

Further, the associations observed in our analysis cannot be interpreted causally without additional assumptions. The temporal order of the priming events considered in our analysis creates a possibility that later exposures could be on a causal pathway between the earlier exposures and the outcomes. We emphasize the hypothesis‐generating nature of the present analysis. Future studies should consider targeted analyses of specific immune‐priming events. Knowing whether receiving an influenza vaccination is protective against COVID‐19 and vice versa would be extremely useful for optimizing vaccination schedules and timing. However, the effect is undoubtedly mediated by a person's unique immunological and exposure history, as well as the relative timing of each immune‐priming event. Potential survival bias arising from restricting the sample to individuals alive on October 1, 2021 (beginning of the outcome period) may limit generalizability but was necessary to reduce misclassification.

Despite the limitations, which are likely to result in conservative estimates of potential protective effects, our study leverages the unique geographically diverse data from over 600,000 people to explore novel and important questions related to immune priming, its timing, and possible crossprotective effects, which have the potential to impact public health policy and clinical practice. The longitudinal nature of the N3C data allowed this analysis to measure associations with less temporally proximate immune‐priming events, which are often not included or available for more controlled study designs.

## 5 Conclusions

This novel exploratory analysis of EHR data from over 600,000 people across seven sites suggests potential crossprotective effects of immune‐priming events on the incidence of viral ARIs, including possible broad protection from COVID‐19 and influenza vaccinations against other pathogens. The strongest negative (i.e., protective) associations between various infection outcomes during October 2021–April 2022 were observed for COVID‐19 vaccination during December 2020–March 2021, off‐season influenza vaccination during January–June 2021 (with the majority vaccinated during January–March 2021) and SARS‐CoV‐2 infection during October 2020–March 2021. The overlap between these temporal windows may suggest the importance of immune priming 6–9 months before the beginning of the respiratory virus season. At the same time, our analysis highlighted the nuances of our research questions and the difficulty of disentangling biological effects from behavioral changes, as well as healthcare‐seeking and reporting patterns. The potential public health effects of confirming whether these associations are causal are substantial, opening an opportunity to increase understanding of severe disease risk and optimize vaccination schedules to further reduce population burden of viral ARIs.

## Author Contributions

All authors had full access to the study data and take responsibility for the integrity of the data and the accuracy of the data analysis. **Tomás M. León:** conceptualization, investigation, funding acquisition, writing – original draft, methodology, writing – review and editing. **Lyndsey M. Muehling:** conceptualization, investigation, funding acquisition, writing – original draft, methodology, writing – review and editing. **Rachel Baccile:** writing – original draft, validation, writing – review and editing, software, formal analysis, data curation. **Olga Morozova:** conceptualization, investigation, funding acquisition, writing – original draft, methodology, visualization, writing – review and editing, formal analysis, project administration, supervision, resources.

## Funding

This project was supported by the National Center for Advancing Translational Sciences (NCATS) of the National Institutes of Health (NIH) through grant number UL1TR002389 that funds the Institute for Translational Medicine (ITM). The content is solely the responsibility of the authors and does not necessarily represent the official views of the NIH. The funders had no role in the design and conduct of the study; management, analysis, and interpretation of the data; preparation and review of the manuscript; and decision to submit the manuscript for publication. Per NIH policy, NCATS approved the final version of the manuscript submitted for publication for compliance with data reporting standards.

## Disclosure

The findings and conclusions in this article are those of the authors and do not necessarily represent the views or opinions of the California Department of Public Health or the California Health and Human Services Agency. The N3C Publication committee confirmed that this manuscript msid: 2623.423 is in accordance with N3C data use and attribution policies; however, this content is solely the responsibility of the authors and does not necessarily represent the official views of the National Institutes of Health or the N3C program.

## Ethics Statement

The N3C data transfer to NCATS is performed under a Johns Hopkins University Reliance Protocol # IRB00249128 or individual site agreements with NIH. The N3C Data Enclave is managed under the authority of the NIH; information can be found at https://ncats.nih.gov/n3c/resources.

## Conflicts of Interest

The authors declare no conflicts of interest.

## Supporting information


**Table S1:** Influenza vaccination coverage by data partner by year.
**Table S2:** URLs for the exposures and outcomes concept sets.
**Table S3:** Immune‐priming events: Counts and proportions (N = 608,725).
**Table S4:** Multivariable logistic regression for the correlates of acute viral respiratory infection outcomes during October 1, 2021–April 30, 2022 in the main sample (N = 608,725).
**Table S5:** Multivariable logistic regression for the correlates of acute viral respiratory infection outcomes during October 1, 2021–April 30, 2022 in the sub‐sample of four sites with highly ascertained influenza vaccination data (N = 213,759).

## Data Availability

The analysis was done using the National Clinical Cohort Collaborative (N3C) COVID‐19 Enclave data, which are available to researchers with an approved protocol and data use request. Data access is governed under the authority of the National Institutes of Health. More information on data access can be found at https://covid.cd2h.org/for‐researchers.

## References

[1] E. J. Chow , T. M. Uyeki , and H. Y. Chu , “The Effects of the COVID‐19 Pandemic on Community Respiratory Virus Activity,” Nature Reviews. Microbiology 21, no. 3 (2023): 195–210, 10.1038/s41579-022-00807-9. 36253478 PMC9574826

[2] CDC , “Preliminary Estimated Flu Disease Burden 2022–2023 Flu Season,” Flu Burden December 9, 2024, accessed July 9, 2025, https://www.cdc.gov/flu‐burden/php/data‐vis/2022‐2023.html.

[3] A. Couture , A. D. Iuliano , H. H. Chang , et al., “State‐Level Influenza Hospitalization Burden in the United States, 2022–2023,” American Journal of Public Health 115, no. 4 (2025): 546–554, 10.2105/AJPH.2024.307928. 39883901 PMC11903080

[4] C. M. Saad‐Roy , S. E. Morris , R. E. Baker , et al., “Medium‐Term Scenarios of COVID‐19 as a Function of Immune Uncertainties and Chronic Disease,” Journal of The Royal Society Interface 20, no. 205 (2023): 20230247, 10.1098/rsif.2023.0247. 37643641 PMC10465195

[5] J. Piret and G. Boivin , “Viral Interference Between Respiratory Viruses,” Emerging Infectious Diseases 28, no. 2 (2022): 273–281, 10.3201/eid2802.211727. 35075991 PMC8798701

[6] S. Gilbert‐Girard , J. Piret , J. Carbonneau , M. Hénaut , N. Goyette , and G. Boivin , “Viral Interference Between Severe Acute Respiratory Syndrome Coronavirus 2 and Influenza A Viruses,” PLoS Pathogens 20, no. 7 (2024): e1012017, 10.1371/journal.ppat.1012017. 39038029 PMC11293641

[7] S. Deleveaux , A. Clarke‐Kregor , X. Fonseca‐Fuentes , and E. Mekhaiel , “Exploring the Possible Phenomenon of Viral Interference Between the Novel Coronavirus and Common Respiratory Viruses,” Journal of Patient‐Centered Research and Reviews 10, no. 2 (2023): 91–97, 10.17294/2330-0698.1995. 37091115 PMC10117529

[8] S. Nickbakhsh , C. Mair , L. Matthews , et al., “Virus–Virus Interactions Impact the Population Dynamics of Influenza and the Common Cold,” Proceedings of the National Academy of Sciences 116, no. 52 (2019): 27142–27150, 10.1073/pnas.1911083116. PMC6936719 31843887

[9] C. M. Moore , E. A. Secor , J. L. Everman , et al., “The Common Cold Is Associated With Protection From SARS‐CoV‐2 Infections,” Journal of Infectious Diseases 232, no. 6 (2025): e920–e930, 10.1093/infdis/jiaf374. 40795882 PMC12718006

[10] J. S. Musuuza , L. Watson , V. Parmasad , N. Putman‐Buehler , L. Christensen , and N. Safdar , “Prevalence and Outcomes of Co‐Infection and Superinfection With SARS‐CoV‐2 and Other Pathogens: A Systematic Review and Meta‐Analysis,” PLoS ONE 16, no. 5 (2021): e0251170, 10.1371/journal.pone.0251170. 33956882 PMC8101968

[11] L. Bai , Y. Zhao , J. Dong , et al., “Coinfection With Influenza A Virus Enhances SARS‐CoV‐2 Infectivity,” Cell Research 31, no. 4 (2021): 395–403, 10.1038/s41422-021-00473-1. 33603116 PMC7890106

[12] W. Chaisawangwong , H. Wang , T. Kouo , et al., “Cross‐Reactivity of SARS‐CoV‐2– and Influenza A–Specific T Cells in Individuals Exposed to SARS‐CoV‐2,” JCI Insight 7, no. 18 (2022): e158308, 10.1172/jci.insight.158308. 36134660 PMC9675569

[13] V. Mysore , X. Cullere , M. L. Settles , et al., “Protective Heterologous T Cell Immunity in COVID‐19 Induced by the Trivalent MMR and Tdap Vaccine Antigens,” Medicus 2, no. 9 (2021): 1050–1071.e7, 10.1016/j.medj.2021.08.004. PMC8363466 34414383

[14] A. Grifoni , D. Weiskopf , S. I. Ramirez , et al., “Targets of T Cell Responses to SARS‐CoV‐2 Coronavirus in Humans With COVID‐19 Disease and Unexposed Individuals,” Cell 181, no. 7 (2020): 1489–1501.e15, 10.1016/j.cell.2020.05.015. 32473127 PMC7237901

[15] J. Mateus , A. Grifoni , A. Tarke , et al., “Selective and Cross‐Reactive SARS‐CoV‐2 T Cell Epitopes in Unexposed Humans,” Science 370, no. 6512 (2020): 89–94, 10.1126/science.abd3871. 32753554 PMC7574914

[16] M. Lipsitch , Y. H. Grad , A. Sette , and S. Crotty , “Cross‐Reactive Memory T Cells and Herd Immunity to SARS‐CoV‐2,” Nature Reviews. Immunology 20, no. 11 (2020): 709–713, 10.1038/s41577-020-00460-4. PMC7537578 33024281

[17] S. Pallikkuth , E. Williams , R. Pahwa , M. Hoffer , and S. Pahwa , “Association of Flu Specific and SARS‐CoV‐2 Specific CD4 T Cell Responses in SARS‐CoV‐2 Infected Asymptomatic Heath Care Workers,” Vaccine 39, no. 41 (2021): 6019–6024, 10.1016/j.vaccine.2021.08.092. 34531078 PMC8403669

[18] P. Aaby , M. G. Netea , and C. S. Benn , “Beneficial Non‐Specific Effects of Live Vaccines Against COVID‐19 and Other Unrelated Infections,” Lancet Infectious Diseases 23, no. 1 (2023): e34–e42, 10.1016/S1473-3099(22)00498-4. 36037824 PMC9417283

[19] R. Sparks , W. W. Lau , C. Liu , et al., “Influenza Vaccination Reveals Sex Dimorphic Imprints of Prior Mild COVID‐19,” Nature 614, no. 7949 (2023): 752–761, 10.1038/s41586-022-05670-5. 36599369 PMC10481789

[20] T. Aydillo , A. Rombauts , D. Stadlbauer , et al., “Immunological Imprinting of the Antibody Response in COVID‐19 Patients,” Nature Communications 12, no. 1 (2021): 3781, 10.1038/s41467-021-23977-1. PMC8213790 34145263

[21] K. Röltgen , S. C. A. Nielsen , O. Silva , et al., “Immune Imprinting, Breadth of Variant Recognition, and Germinal Center Response in Human SARS‐CoV‐2 Infection and Vaccination,” Cell 185, no. 6 (2022): 1025–1040.e14, 10.1016/j.cell.2022.01.018. 35148837 PMC8786601

[22] C. P. Arevalo , V. Le Sage , M. J. Bolton , et al., “Original Antigenic Sin Priming of Influenza Virus Hemagglutinin Stalk Antibodies,” Proceedings of the National Academy of Sciences of the United States of America 117, no. 29 (2020): 17221–17227, 10.1073/pnas.1920321117. 32631992 PMC7382271

[23] C. Phetsouphanh , D. R. Darley , D. B. Wilson , et al., “Immunological Dysfunction Persists for 8 Months Following Initial Mild‐to‐Moderate SARS‐CoV‐2 Infection,” Nature Immunology 23, no. 2 (2022): 210–216, 10.1038/s41590-021-01113-x. 35027728

[24] J. Klein , J. Wood , J. R. Jaycox , et al., “Distinguishing Features of Long COVID Identified Through Immune Profiling,” Nature 623, no. 7985 (2023): 139–148, 10.1038/s41586-023-06651-y. 37748514 PMC10620090

[25] G. Canderan , L. M. Muehling , A. Kadl , et al., “Distinct Type 1 Immune Networks Underlie the Severity of Restrictive Lung Disease After COVID‐19,” Nature Immunology 26, no. 4 (2025): 595–606, 10.1038/s41590-025-02110-0. 40140496 PMC12169215

[26] H. A. Shuwa , T. N. Shaw , S. B. Knight , et al., “Alterations in T and B Cell Function Persist in Convalescent COVID‐19 Patients,” Medicus 2, no. 6 (2021): 720–735.e4, 10.1016/j.medj.2021.03.013. PMC8011689 33821250

[27] D. Damjanovic , C. L. Small , M. Jeyananthan , S. McCormick , and Z. Xing , “Immunopathology in Influenza Virus Infection: Uncoupling the Friend From Foe,” Clinical Immunology 144, no. 1 (2012): 57–69, 10.1016/j.clim.2012.05.005. 22673491

[28] S. Y. Kim , J. H. Kim , M. Kim , et al., “The Associations of Previous Influenza/Upper Respiratory Infection With COVID‐19 Susceptibility/Morbidity/Mortality: A Nationwide Cohort Study in South Korea,” Scientific Reports 11, no. 1 (2021): 21568, 10.1038/s41598-021-00428-x. 34732751 PMC8566493

[29] B. J. Cowling , V. J. Fang , H. Nishiura , et al., “Increased Risk of Noninfluenza Respiratory Virus Infections Associated With Receipt of Inactivated Influenza Vaccine,” Clinical Infectious Diseases 54, no. 12 (2012): 1778–1783. 22423139 10.1093/cid/cis307 PMC3404712

[30] S. Rikin , H. Jia , C. Y. Vargas , et al., “Assessment of Temporally‐Related Acute Respiratory Illness Following Influenza Vaccination,” Vaccine 36, no. 15 (2018): 1958–1964, 10.1016/j.vaccine.2018.02.105. 29525279 PMC7115556

[31] A. Dierig , L. G. Heron , S. B. Lambert , et al., “Epidemiology of Respiratory Viral Infections in Children Enrolled in a Study of Influenza Vaccine Effectiveness,” Influenza and Other Respiratory Viruses 8, no. 3 (2014): 293–301, 10.1111/irv.12229. 24483149 PMC4181477

[32] M. E. Sundaram , D. L. McClure , J. J. VanWormer , T. C. Friedrich , J. K. Meece , and E. A. Belongia , “Influenza Vaccination Is Not Associated With Detection of Noninfluenza Respiratory Viruses in Seasonal Studies of Influenza Vaccine Effectiveness,” Clinical Infectious Diseases 57, no. 6 (2013): 789–793, 10.1093/cid/cit379. 23748138 PMC7107973

[33] S. Feng , A. L. Fowlkes , A. Steffens , L. Finelli , and B. J. Cowling , “Assessment of Virus Interference in a Test‐Negative Study of Influenza Vaccine Effectiveness,” Epidemiology 28, no. 4 (2017): 514–524, 10.1097/EDE.0000000000000670. 28362642 PMC5535302

[34] R. P. Jones , A. Ponomarenko , R. P. Jones , and A. Ponomarenko , “System Complexity in Influenza Infection and Vaccination: Effects Upon Excess Winter Mortality,” Infectious Disease Reports 14, no. 3 (2022): 287–309, 10.3390/idr14030035. 35645214 PMC9149983

[35] A. Conlon , C. Ashur , L. Washer , K. A. Eagle , and M. A. Hofmann Bowman , “Impact of the Influenza Vaccine on COVID‐19 Infection Rates and Severity,” American Journal of Infection Control 49, no. 6 (2021): 694–700, 10.1016/j.ajic.2021.02.012. 33631305 PMC7899024

[36] Data Transfer Agreement Signatories| National Center for Advancing Translational Sciences , accessed July 22, 2025, https://ncats.nih.gov/research/research‐activities/n3c/resources/data‐contribution/signatories.

[37] M. A. Haendel , C. G. Chute , T. D. Bennett , et al., “The National COVID Cohort Collaborative (N3C): Rationale, Design, Infrastructure, and Deployment,” Journal of the American Medical Informatics Association 28, no. 3 (2021): 427–443, 10.1093/jamia/ocaa196. 32805036 PMC7454687

[38] National Clinical Cohort Collaborative , “COVID‐19 Phenotype Documentation,” GitHub, accessed July 22, 2025, https://github.com/National‐Clinical‐Cohort‐Collaborative/Phenotype_Data_Acquisition/wiki/Latest‐Phenotype.

[39] S. O'Neil and W. H. Beasley . 2023. Guide to N3C (v0.4). Zenodo, 10.5281/zenodo.7749367.

[40] M. D. Brannock , R. F. Chew , A. J. Preiss , et al., “Long COVID Risk and Pre‐COVID Vaccination in an EHR‐Based Cohort Study From the RECOVER Program,” Nature Communications 14, no. 1 (2023): 2914, 10.1038/s41467-023-38388-7. PMC10201472 37217471

[41] A. C. Cameron and D. L. Miller , “A Practitioner's Guide to Cluster‐Robust Inference,” Journal of Human Resources 50, no. 2 (2015): 317–372, 10.3368/jhr.50.2.317.

[42] H. Quan , B. Li , C. M. Couris , et al., “Updating and Validating the Charlson Comorbidity Index and Score for Risk Adjustment in Hospital Discharge Abstracts Using Data From 6 Countries,” American Journal of Epidemiology 173, no. 6 (2011): 676–682, 10.1093/aje/kwq433. 21330339

[43] E. von Elm , D. G. Altman , M. Egger , S. J. Pocock , P. C. Gøtzsche , and J. P. Vandenbroucke , “The Strengthening the Reporting of Observational Studies in Epidemiology (STROBE) Statement: Guidelines for Reporting Observational Studies,” Lancet 370, no. 9596 (2007): 1453–1457, 10.1016/S0140-6736(07)61602-X. 18064739

[44] C. Graña , L. Ghosn , T. Evrenoglou , et al., “Efficacy and Safety of COVID‐19 Vaccines,” Cochrane Database of Systematic Reviews 12, no. 12 (2022): CD015477, 10.1002/14651858.CD015477. 36473651 PMC9726273

[45] G. Zhou , N. Dael , S. Verweij , et al., “Effectiveness of COVID‐19 Vaccines Against SARS‐CoV‐2 Infection and Severe Outcomes in Adults: A Systematic Review and Meta‐Analysis of European Studies Published Up to 22 January 2024,” European Respiratory Review 34, no. 175 (2025): 240222, 10.1183/16000617.0222-2024. 39971395 PMC11836669

[46] T. K. Tsang , S. G. Sullivan , X. Huang , et al., “Prior Infections and Effectiveness of SARS‐CoV‐2 Vaccine in Test‐Negative Studies: A Systematic Review and Meta‐Analysis,” American Journal of Epidemiology 193, no. 12 (2024): 1868–1881, 10.1093/aje/kwae142. 38904437 PMC11637527

[47] S. J. Smart and S. W. Polachek , “COVID‐19 Vaccine and Risk‐Taking,” Journal of Risk and Uncertainty 68, no. 1 (2024): 25–49, 10.1007/s11166-023-09424-0.

[48] J. Liu , B. Kassas , J. Lai , J. Kropp , and Z. Gao , “Understanding the Role of Risk Preferences and Perceptions in Vaccination Decisions and Post‐Vaccination Behaviors among U.S. Households,” Scientific Reports 14, no. 1 (2024): 3190, 10.1038/s41598-024-52408-6. 38326338 PMC10850518

[49] N. Andrews , J. Stowe , F. Kirsebom , et al., “Covid‐19 Vaccine Effectiveness Against the Omicron (B.1.1.529) Variant,” New England Journal of Medicine 386, no. 16 (2022): 1532–1546, 10.1056/NEJMoa2119451. 35249272 PMC8908811

[50] D. Y. Lin , Y. Gu , B. Wheeler , et al., “Effectiveness of Covid‐19 Vaccines Over a 9‐Month Period in North Carolina,” New England Journal of Medicine 386, no. 10 (2022): 933–941, 10.1056/NEJMoa2117128. 35020982 PMC8781317

[51] Y. Goldberg , M. Mandel , Y. M. Bar‐On , et al., “Protection and Waning of Natural and Hybrid Immunity to SARS‐CoV‐2,” New England Journal of Medicine 386, no. 23 (2022): 2201–2212, 10.1056/NEJMoa2118946. 35613036 PMC9165562

[52] W. W. Viscusi , “The Lulling Effect: The Impact of Child‐Resistant Packaging on Aspirin and Analgesic Ingestions,” American Economic Review 74, no. 2 (1984): 324–327. 11616492

[53] J. C. Taube , Z. Susswein , and S. Bansal , “Spatiotemporal Trends in Self‐Reported Mask‐Wearing Behavior in the United States: Analysis of a Large Cross‐Sectional Survey,” JMIR Public Health and Surveillance 9 (2023): e42128, 10.2196/42128. 36877548 PMC10028521

[54] A. H. Tjaden , M. Gibbs , M. Runyon , W. S. Weintraub , Y. J. Taylor , and S. L. Edelstein , “Association Between Self‐Reported Masking Behavior and SARS‐CoV‐2 Infection Wanes From Pre‐Delta to Omicron‐Predominant Periods — North Carolina COVID‐19 Community Research Partnership (NC‐CCRP),” American Journal of Infection Control 51, no. 3 (2023): 261–267, 10.1016/j.ajic.2022.09.027. 36209944 PMC9537112

[55] H. Chemaitelly , H. H. Ayoub , P. Coyle , et al., “Differential Protection Against SARS‐CoV‐2 Reinfection Pre‐ and Post‐Omicron,” Nature 639, no. 8056 (2025): 1024–1031, 10.1038/s41586-024-08511-9. 39910292 PMC11946897

[56] S. Carazo , J. Phimmasone , D. M. Skowronski , et al., “Effectiveness of COVID‐19 Vaccination and Prior Infections to Reduce Long COVID Risk During the Pre‐Omicron and Omicron Periods,” Clinical Infectious Diseases. Published online September 29 (2025): ciaf549, 10.1093/cid/ciaf549. PMC13016730 41021660

[57] C. F. Wei , F. Y. Lan , Y. T. Hsu , et al., “Risk of SARS‐CoV‐2 Infection Among Essential Workers in a Community‐Based Cohort in the United States,” Frontiers in Public Health 10 (2022): 878208, 10.3389/fpubh.2022.878208. 35677773 PMC9169416

[58] S. W. X. Ong , C. J. Chiew , L. W. Ang , et al., “Clinical and Virological Features of Severe Acute Respiratory Syndrome Coronavirus 2 (SARS‐CoV‐2) Variants of Concern: A Retrospective Cohort Study Comparing B.1.1.7 (Alpha), B.1.351 (Beta), and B.1.617.2 (Delta),” Clinical Infectious Diseases 75, no. 1 (2022): e1128–e1136, 10.1093/cid/ciab721. 34423834 PMC8522361

[59] K. T. Copeland , H. Checkoway , A. J. McMichael , and R. H. Holbrook , “Bias Due to Misclassification in the Estimation of Relative Risk,” American Journal of Epidemiology 105, no. 5 (1977): 488–495, 10.1093/oxfordjournals.aje.a112408. 871121

[60] J. Harton , N. Mitra , and R. A. Hubbard , “Informative Presence Bias in Analyses of Electronic Health Records‐Derived Data: A Cautionary Note,” Journal of the American Medical Informatics Association 29, no. 7 (2022): 1191–1199, 10.1093/jamia/ocac050. 35438796 PMC9196698

[61] S. G. Sullivan , E. J. Tchetgen Tchetgen , and B. J. Cowling , “Theoretical Basis of the Test‐Negative Study Design for Assessment of Influenza Vaccine Effectiveness,” American Journal of Epidemiology 184, no. 5 (2016): 345–353, 10.1093/aje/kww064. 27587721 PMC5013887

[62] M. Valenciano , B. C. Ciancio , and A. Moren , “The Influenza Vaccine Effectiveness Working Group. First Steps in the Design of a System to Monitor Vaccine Effectiveness During Seasonal and Pandemic Influenza in EU/EEA Member States,” Eurosurveillance 13, no. 43 (2008): 19015, 10.2807/ese.13.43.19015-en. 18947520

[63] A. M. Parker , S. Atshan , M. M. Walsh , C. A. Gidengil , and R. Vardavas , “Association of COVID‐19 Vaccination With Influenza Vaccine History and Changes in Influenza Vaccination,” JAMA Network Open 5, no. 11 (2022): e2241888, 10.1001/jamanetworkopen.2022.41888. 36374504 PMC9664264

