# Supplementary material for: Immune Priming and the Risk of COVID‐19, Influenza, and Other Acute Respiratory Infections: Insights From an N3C Cohort
Source: Influenza Other Respir Viruses. 2026 Mar 23;20(3):e70253. doi: 10.1111/irv.70253 (PMC13098052; doi:10.1111/irv.70253)
Supplement: Supplementary file 1 — Table S1: Influenza vaccination coverage by data partner by year. Table S2: URLs for the exposures and outcomes concept sets. Table S3: Immune‐priming events: Counts and proportions (N = 608,725). Table S4: Multivariable logistic regression for the correlates of acute viral respiratory infection outcomes during October 1, 2021–April 30, 2022 in the main sample (N = 608,725). Table S5: Multivariable logistic regression for the correlates of acute viral respiratory infection outcomes during October 1, 2021–April 30, 2022 in the sub‐sample of four sites with highly ascertained influenza vaccination data (N = 213,759). [file IRV-20-e70253-s001.pdf]

Supplementary Appendix to

Immune Priming and the Risk of COVID-19, Influenza, and Other Acute Respiratory Infections: Insights From an N3C Cohort

Authors: Tomás M. León*[1]; Lyndsey M. Muehling*[2]; Rachel Baccile[3]; Olga Morozova[4]; National Clinical Cohort Collaborative (N3) COVID-19 Consortium

[1] California Department of Public Health, Richmond, CA, United States

[2] Division of Asthma, Allergy & Immunology, University of Virginia School of Medicine, Charlottesville, VA, United States

[3] Center for Health and Social Sciences, University of Chicago, Chicago, IL, United States

[4] Department of Public Health Sciences, University of Chicago, Chicago, IL, United States

* Tomás M. León and Lyndsey M. Muehling contributed equally to this study.

Supplementary Table S1. Influenza vaccination coverage by data partner by year.

Supplementary Table S2. URLs for the exposures and outcomes concept sets.

Supplementary Table S3. Immune priming events: Counts and proportions (N=608,725).

Supplementary Table S4. Multivariable logistic regression for the correlates of acute viral respiratory infection outcomes during October 1, 2021 – April 30, 2022 in the main sample (N=608,725).

Supplementary Table S5. Multivariable logistic regression for the correlates of acute viral respiratory infection outcomes during October 1, 2021 – April 30, 2022 in the sub-sample of four sites with highly ascertained influenza vaccination data (N=213,759).

Supplementary Table S1. Influenza vaccination coverage by data partner by year.

Data Partner ID (masked) / Influenza season*	Influenza Vaccinations: % of Patients at the DP	Average Influenza Vaccinations (2018-2020): % of Patients at the DP	Inclusion in the Analytic Sample (Average Influenza Vaccinations >25%)
DP 1		71.8%	Included
2018	80.8%		
2019	74.5%		
2020	60.1%		
DP 2		66.0%	Included
2018	77.7%		
2019	68.5%		
2020	51.9%		
DP 3		61.0%	Included
2018	68.5%		
2019	63.8%		
2020	50.7%		
DP 4		59.0%	Included
2018	70.7%		
2019	59.0%		
2020	47.2%		
DP 5		31.6%	Included
2018	40.9%		
2019	30.8%		
2020	22.9%		
DP 6		26.4%	Included
2018	35.7%		
2019	33.4%		
2020	10.2%		
DP 7		31.5%	Included
2018	35.8%		
2019	36.9%		
2020	22.0%		
DP 8		2.2%	Excluded
2018	4.3%		
2019	1.6%		
2020	0.8%		
DP 9		12.0%	Excluded
2018	17.7%		
2019	11.8%		
2020	6.4%		
DP 10		18.5%	Excluded
2018	22.2%		
2019	18.5%		
2020	14.7%		
DP 11		17.0%	Excluded
2018	19.8%		
2019	15.1%		
2020	16.1%		
DP 12		12.6%	Excluded
2018	12.2%		
2019	11.6%		
2020	13.9%		

* The following date ranges are used for annual influenza seasons: 2018: 7/1/2018 – 6/30/2019; 2019: 7/1/2019 – 6/30/2020; 2020: 7/1/2020 – 6/30/2021.

Supplementary Table S2. N3C Enclave URLs for the exposures, outcomes and Charlson Comorbidity Index concept sets.

Concept Set	N3C Enclave URL
SARS-CoV-2 infection (standard N3C definition)	https://unite.nih.gov/workspace/hubble/objects/ri.phonograph2-objects.main.object.b826e8b4-30e9-49ef-9f00-1c5e84357910; https://unite.nih.gov/workspace/hubble/objects/ri.phonograph2-objects.main.object.48db2a56-e02b-46f2-9660-315532f0e9e6
COVID-19 vaccination template (standard N3C definition)	https://unite.nih.gov/workspace/module/view/latest/ri.workshop.main.module.3ab34203-d7f3-482e-adbd-f4113bfd1a2b?id=KO-4BE516B&view=focus
Influenza infection	https://unite.nih.gov/workspace/hubble/objects/ri.phonograph2-objects.main.object.f23099a7-33d4-4bab-a918-08335c82c5d5
Influenza vaccination	https://unite.nih.gov/workspace/hubble/objects/ri.phonograph2-objects.main.object.2951936a-7335-4014-9141-194985613c6c
Respiratory syncytial virus (RSV)	https://unite.nih.gov/workspace/hubble/objects/ri.phonograph2-objects.main.object.9fe1e311-c302-4dc1-a438-6ce9f81f5096
Other acute viral respiratory infection	https://unite.nih.gov/workspace/hubble/objects/ri.phonograph2-objects.main.object.78b20ebd-d1ee-4788-936a-7676aee4d90f
Unspecified acute viral respiratory infection	https://unite.nih.gov/workspace/hubble/objects/ri.phonograph2-objects.main.object.99e30300-9c14-4330-a72c-c7451d457d6e
Charlson Comorbidity Index Template*	https://unite.nih.gov/workspace/module/view/latest/ri.workshop.main.module.3ab34203-d7f3-482e-adbd-f4113bfd1a2b?id=KO-06D5DA0&view=focus

The spreadsheet versions of the concept sets outside of the N3C enclave are available at: https://github.com/olyamorozova/N3C-ARI-concepts.git

* The Charlson Comorbidity Index (CCI) is a weighted score that encompasses the following 17 clinical conditions:

- Myocardial infarction
- Congestive heart failure
- Peripheral vascular disease
- Cerebrovascular disease
- Hemiplegia or paraplegia
- Dementia
- Chronic pulmonary disease
- Rheumatologic disease
- Peptic ulcer disease
- Diabetes without chronic complications
- Diabetes with chronic complications
- Renal disease
- Any malignancy, including leukemia and lymphoma
- Metastatic solid tumor
- Mild liver disease
- Moderate or severe liver disease
- HIV

For details on the CCI definition, see: Quan H, Li B, Couris CM, et al. Updating and Validating the Charlson Comorbidity Index and Score for Risk Adjustment in Hospital Discharge Abstracts Using Data From 6 Countries. *American Journal of Epidemiology*. 2011;173(6):676-682. doi:10.1093/aje/kwq433.

Supplementary Table S3. Immune priming events: Counts and proportions (N=608,725).

Event	N	%
COVID-19 vaccination		
December 2020 – March 2021	285,140	46.8%
April – June 2021	198,414	32.6%
July – September 2021	74,708	12.3%
SARS-CoV-2 infection		
January – September 2020	28,719	4.7%
October 2020 – March 2021	58,651	9.6%
April – September 2021	24,112	4.0%
Influenza vaccination		
January 2018 – June 2019	170,477	28.0%
July 2019 – June 2020	174,293	28.6%
July – December 2020	153,205	25.2%
January – June 2021	10,391	1.7%
July – September 2021	39,075	6.4%
Influenza infection		
January 2018 – September 2019	8,280	1.4%
October 2019 – September 2020	10,180	1.7%
October 2020 – March 2021	3,037	0.5%
April – September 2021	739	0.1%
Other respiratory viral infections, including unspecified		
January 2018 – September 2019	33,064	5.4%
October 2019 – September 2020	24,389	4.0%
October 2020 – March 2021	6,112	1.0%
April – September 2021	9,919	1.6%

Supplementary Table S4. Multivariable logistic regression for the correlates of acute viral respiratory infection outcomes during October 1, 2021 – April 30, 2022 in the main sample (N=608,725).

Immune priming event	Any acute viral respiratory infection		Any infection excluding SARS-CoV-2		SARS-CoV-2		Influenza	
	aOR	95% CI	aOR	95% CI	aOR	95% CI	aOR	95% CI
COVID-19 vaccination								
December 2020 – March 2021	0.77	(0.67; 0.88)	0.86	(0.81; 0.91)	0.74	(0.62; 0.88)	0.83	(0.79; 0.88)
April – June 2021	0.70	(0.63; 0.78)	0.97	(0.91; 1.04)	0.64	(0.55; 0.73)	0.96	(0.87; 1.06)
July – September 2021	0.87	(0.78; 0.96)	0.95	(0.88; 1.03)	0.85	(0.74; 0.97)	0.97	(0.88; 1.07)
SARS-CoV-2 infection								
January – September 2020	0.61	(0.39; 0.94)	0.87	(0.73; 1.04)	0.55	(0.29; 1.05)	1.07	(0.75; 1.52)
October 2020 – March 2021	0.60	(0.45; 0.80)	0.86	(0.77; 0.96)	0.53	(0.34; 0.81)	0.88	(0.79; 0.98)
April – September 2021	1.03	(0.74; 1.43)	0.93	(0.86; 1.00)	1.06	(0.71; 1.58)	1.00	(0.86; 1.15)
Influenza vaccination								
January 2018 – June 2019	0.97	(0.9; 1.04)	1.04	(1.01; 1.07)	0.95	(0.87; 1.04)	1.03	(0.97; 1.10)
July 2019 – June 2020	0.91	(0.83; 1.00)	0.95	(0.89; 1.01)	0.90	(0.81; 0.99)	0.94	(0.92; 0.96)
July – December 2020	0.94	(0.83; 1.06)	1.01	(0.97; 1.04)	0.92	(0.78; 1.08)	0.91	(0.78; 1.07)
January – June 2021	0.78	(0.74; 0.84)	1.01	(0.94; 1.08)	0.73	(0.69; 0.78)	0.91	(0.76; 1.10)
July – September 2021	1.13	(1.02; 1.26)	1.04	(0.99; 1.09)	1.14	(1.02; 1.28)	0.92	(0.79; 1.08)
Influenza infection								
January 2018 – September 2019	1.26	(1.18; 1.34)	1.35	(1.20; 1.52)	1.21	(1.11; 1.32)	1.44	(1.09; 1.89)
October 2019 – September 2020	1.18	(1.08; 1.28)	1.40	(1.31; 1.49)	1.07	(0.96; 1.18)	1.54	(1.42; 1.67)
October 2020 – March 2021	1.07	(0.87; 1.32)	1.72	(1.37; 2.15)	0.88	(0.72; 1.08)	2.17	(1.77; 2.66)
April – September 2021	1.27	(0.81; 2.00)	2.30	(1.90; 2.80)	0.98	(0.62; 1.55)	3.13	(2.44; 4.01)
Other respiratory viral infections, including unspecified								
January 2018 – September 2019	1.41	(1.28; 1.56)	1.79	(1.62; 1.97)	1.18	(1.12; 1.25)	1.63	(1.44; 1.84)
October 2019 – September 2020	1.29	(1.18; 1.41)	1.70	(1.61; 1.79)	1.06	(0.95; 1.18)	1.28	(1.11; 1.48)
October 2020 – March 2021	1.50	(1.27; 1.78)	2.25	(2.07; 2.44)	1.03	(0.92; 1.16)	1.53	(1.05; 2.21)
April – September 2021	1.68	(1.37; 2.07)	2.53	(2.24; 2.86)	1.15	(1.02; 1.30)	1.83	(1.59; 2.10)

All regression models are adjusted for age, sex, race/ethnicity, Charlson Comorbidity Index, data partner and log of the number of healthcare encounters between January 1, 2020 – April 30, 2022.

Supplementary Table S5. Multivariable logistic regression for the correlates of acute viral respiratory infection outcomes during October 1, 2021 – April 30, 2022 in the sub-sample of four sites with highly ascertained influenza vaccination data (N=213,759).

Immune priming event	Any acute viral respiratory infection		Any infection excluding SARS-CoV-2		SARS-CoV-2		Influenza	
	aOR	95% CI	aOR	95% CI	aOR	95% CI	aOR	95% CI
COVID-19 vaccination								
December 2020 – March 2021	0.84	(0.63; 1.11)	0.94	(0.80; 1.10)	0.83	(0.61; 1.13)	0.95	(0.86; 1.05)
April – June 2021	0.72	(0.57; 0.93)	1.10	(0.99; 1.22)	0.70	(0.55; 0.89)	1.09	(0.89; 1.35)
July – September 2021	0.93	(0.78; 1.11)	0.98	(0.86; 1.12)	0.93	(0.76; 1.13)	1.06	(0.77; 1.46)
SARS-CoV-2 infection								
January – September 2020	0.77	(0.32; 1.86)	0.72	(0.59; 0.88)	0.78	(0.29; 2.08)	0.64	(0.37; 1.08)
October 2020 – March 2021	0.66	(0.34; 1.31)	0.96	(0.85; 1.08)	0.62	(0.28; 1.41)	0.74	(0.59; 0.94)
April – September 2021	1.10	(0.53; 2.29)	0.98	(0.87; 1.11)	1.11	(0.50; 2.47)	1.00	(0.65; 1.54)
Influenza vaccination								
January 2018 – June 2019	1.00	(0.87; 1.14)	1.08	(0.97; 1.22)	0.99	(0.86; 1.13)	1.17	(1.08; 1.27)
July 2019 – June 2020	0.94	(0.84; 1.06)	0.98	(0.92; 1.04)	0.94	(0.83; 1.07)	0.94	(0.90; 0.99)
July – December 2020	0.97	(0.78; 1.22)	1.01	(0.88; 1.16)	0.97	(0.77; 1.23)	1.01	(0.73; 1.39)
January – June 2021	0.79	(0.74; 0.83)	1.01	(0.81; 1.25)	0.78	(0.74; 0.82)	0.79	(0.72; 0.86)
July – September 2021	1.11	(0.92; 1.35)	0.96	(0.90; 1.03)	1.12	(0.93; 1.35)	0.76	(0.60; 0.96)
Influenza infection								
January 2018 – September 2019	1.33	(1.16; 1.53)	1.48	(1.20; 1.83)	1.32	(1.14; 1.54)	1.86	(1.43; 2.43)
October 2019 – September 2020	1.13	(0.90; 1.41)	1.54	(1.30; 1.82)	1.02	(0.83; 1.26)	1.64	(1.41; 1.91)
October 2020 – March 2021	1.02	(0.58; 1.79)	2.02	(1.04; 3.93)	0.78	(0.51; 1.20)	2.78	(1.80; 4.28)
April – September 2021	1.04	(0.69; 1.58)	2.65	(1.73; 4.06)	0.76	(0.57; 1.01)	3.75	(3.10; 4.53)
Other respiratory viral infections, including unspecified								
January 2018 – September 2019	1.33	(1.20; 1.48)	1.92	(1.71; 2.15)	1.21	(1.13; 1.30)	1.79	(1.40; 2.31)
October 2019 – September 2020	1.31	(1.13; 1.51)	1.98	(1.71; 2.29)	1.16	(1.01; 1.33)	1.52	(1.40; 1.64)
October 2020 – March 2021	1.26	(0.87; 1.81)	2.45	(1.85; 3.26)	0.96	(0.71; 1.30)	1.62	(1.10; 2.37)
April – September 2021	1.46	(1.12; 1.89)	2.73	(2.00; 3.72)	1.19	(0.96; 1.47)	1.39	(1.02; 1.88)

All regression models are adjusted for age, sex, race/ethnicity, Charlson Comorbidity Index, data partner and log of the number of healthcare encounters between January 1, 2020 – April 30, 2022.
